# The Impact of Parental Engagement in an Electronic Health (EHealth) Intervention on Physical Activity, Dietary Behaviors, and Sleep in Preschool-Aged Children

**DOI:** 10.3390/ijerph23030345

**Published:** 2026-03-10

**Authors:** Peng Zhou, Wenjiao Liu, Di Li

**Affiliations:** 1 Department of Sports and Recreation, Technological and Higher Education Institute of Hong Kong, Hong Kong, China; bookerzp@thei.edu.hk; 2 Department of Education, UCSI University, Kuala Lumpur 56000, Malaysia; 3 Department of Chinese Language Studies, Education University of Hong Kong, New Territories, Hong Kong, China; s1146948@s.eduhk.hk

**Keywords:** 24-h movement behaviors, parenting practices, preschoolers

## Abstract

**Highlights:**

**Public health relevance—How does this work relate to a public health issue?**

- Targeting Early Childhood Obesity Risks: This work addresses critical lifestyle behaviors—physical activity, diet, and sedentary screen time—in preschoolers (aged 3–6), a developmental window essential for establishing lifelong health habits and preventing chronic disease.
- Leveraging Digital Health (eHealth) Platforms: It explores the utility of ubiquitous social media platforms (like WeChat) as a delivery mechanism for parent-based interventions, reflecting the modern shift toward scalable, technology-driven public health strategies.

**Public health significance—Why is this work of significance to public health?**

- Identifying the “Engagement Gap”: The study highlights that the *quality* of parental participation (active posting vs. “lurking”) is a significant determinant of intervention success, suggesting that simply providing digital content is insufficient for behavior change.
- Impact on Multi-Domain Health Outcomes: The findings demonstrate that active parental engagement specifically correlates with improved physical activity intensity and better dietary behaviors (reduced food fussiness and satiety responsiveness), offering a clear pathway to more effective childhood health programs.

**Public health implications—What are the key implications or messages for practitioners, policy makers and/or researchers in public health?**

- For Practitioners: Interventionists should move beyond “one-size-fits-all” digital delivery and develop specific strategies to convert “lurkers” into active participants to ensure the intended health benefits are realized by the children.
- For Policy Makers and Researchers: Future public health policies and research should prioritize the design of interactive, socially driven eHealth features that foster active engagement, rather than passive consumption, to maximize the return on investment for digital health initiatives.

**Abstract:**

**Background/Objectives**: The characterization of varying levels of parental engagement is important for increasing understanding of how to tailor and maximize the effectiveness of parent-based eHealth interventions. In this study, we aimed to determine if parental engagement in the WeChat group of a parent-based eHealth intervention affected preschoolers’ physical activity, diet, or sleep. **Methods**: We utilized baseline, post-test (12 weeks after baseline), and follow-up (12 weeks after post-test measurement) data from the intervention group in a parent-based eHealth intervention concerning children aged from three to six years, designed as a single-blinded randomized controlled trial with two parallel arms to explore the intervention’s influence on preschoolers’ physical activity, diet, and sleep. The parents in the intervention group were categorized into two groups: (1) The actively engaged group (53 parent–child dyads), defined as parents who actively posted and commented on modules at least once a week, either in the WeChat groups or through private messages with the researchers. (2) The lurker group (67 parent–child dyads), defined as parents who only responded to the weekly self-assessment messages and who, aside from this, showed no interaction within WeChat groups and did not privately message the researchers. Preschoolers’ physical activity was measured using ActiGraph wGT3X-BT, while their dietary behaviors and sleep were measured using parent-reported questionnaires. Generalized Estimating Equations using group and time as main effects and adjusted demographic information for covariates were computed to examine the effects of parental engagement in the eHealth intervention on preschoolers’ physical activity, diet, and sleep. **Results**: At post-test, higher levels of parental engagement were significantly associated with a marked increase in preschoolers’ moderate-to-vigorous and vigorous physical activity, alongside a notable reduction in weekend screen time. Furthermore, active parental engagement was linked to greater decreases in satiety responsiveness, desire to drink, and food fussiness compared to the ‘lurker’ group. However, no significant associations were observed between the level of parental engagement and preschoolers’ sleep-related outcomes. **Conclusions**: Further research with larger sample sizes and longer durations is needed to better investigate the potential of social media in parent-based interventions for promoting healthy lifestyles in children.

## 1. Introduction

Preschoolers are becoming more physically inactive [1], engaging in sedentary behaviors including recreational screen time [2], adopting unhealthy eating habits [3], and experiencing greater disruptions in their sleep routines [4]. Establishing these unhealthy lifestyle behaviors at an early age not only triggers health-related issues (e.g., metabolic and musculoskeletal disorders, depression, and behavioral problems) [5,6,7], but means they are more likely to be maintained throughout the lifetime [8]. This emphasizes the importance of cultivating healthy lifestyle behaviors, including physical activity (PA), dietary behavior (DB), and sleep, in early childhood, when behavior patterns are malleable and less entrenched [9].

Social cognitive theory (SCT) proposes that learning occurs as a social phenomenon through interactions with others, primarily by observing and watching [10]. Young children are primarily socialized by their parents, while also being enrolled in early childhood education centers, such as kindergartens [11,12], with previous studies aimed at improving preschoolers’ lifestyle behaviors identifying both environments as critical. Given that up to 90% of preschoolers attend kindergartens [13], previous studies initially focused on school-based face-to-face interventions to improve preschoolers’ PA, but they encountered several challenges: (1) high staff turnover rates in kindergartens made intervention implementation difficult [14]; (2) teachers were often reluctant to participate, as interventions detracted from other educational activities [15]; and (3) differences in individual teaching styles and interpretations of interventions resulted in inconsistent adherence to intervention protocols [16]. As a result, emphasis has shifted to parent-based face-to-face interventions, which still included some recognized barriers (e.g., scheduling of appointments with parents, childcare for other siblings, and travel-related costs) preventing parental engagement and potentially hindering intervention success [17,18,19,20]. In response, focus has moved to parent-based electronic health (eHealth) interventions. eHealth—defined as the cost-effective and secure application of information and communication technologies to support health, including healthcare services, health monitoring, health literature, education, knowledge, and research—has the potential to overcome these barriers [21]. It offers convenience and flexibility for parents, allowing participation regardless of their geographic location [22], and the personalized information and feedback provided through eHealth interventions can enhance strategies for behavioral change [23].

A previous systematic review by Zhou, Li [24], who evaluated the effects of parent-based eHealth intervention on PA, DB, and sleep in preschoolers across twelve eligible studies, found that, in comparison with control groups post-intervention, (1) in the PA-related studies reviewed (7/12), PA was not enhanced in parent-based eHealth interventions; (2) in the DB-related studies reviewed (8/12), significant improvements were observed in increased fruit and vegetable consumption and decreased sweetened beverage, candy, and non-core food consumption; and (3) in the sleep-related studies reviewed (4/12), sleep duration was significantly promoted, but there were no significant changes in sleep problems. These results can be explained as follows. Firstly, the existing literature examining the impact of parent-based eHealth intervention on preschoolers’ PA, DB, and sleep has not balanced their intervention modules (i.e., DB-related modules are more extensive than PA- and sleep-related ones). For example, Hammersleyet al. [25] deployed only one PA-related module (diet*2, sleep*1, screen time*1) during the 11-week intervention period, and Knowlden, Sharma [26] delivered only one (vs. diet*2, screen time*1) in a 4-week period, while Nyström, Sandin [27] implemented only one PA-related module (diet*10, sleep*1) in a 6-month period. It seems possible that fewer PA- and sleep-related modules including knowledge, group interaction, goal setting, tailored feedback, and weekly goal revision may draw the attention of fewer parents in the intervention group, potentially limiting the support parents give to their children regarding PA and sleep [28]. Additionally, Zhou, Li [24] noted that the order of module delivery was overlooked, as parents might have been more familiar with the later-distributed modules during the post-test, resulting in more favorable outcomes for those delivered late on compared to earlier ones. A previous study further concluded that as parenting practices are profoundly shaped by cultural context, these results based upon data from Western countries might not be applicable worldwide [29].

Accordingly, Peng, Wen [30] conducted a randomized controlled trial (RCT) with balanced intervention module content and delivery sequences to assess the effectiveness of parent-based eHealth interventions targeting PA, DB, and sleep in preschoolers in China. The main analyses found that educating parents about healthy lifestyles through social media effectively enhanced preschoolers’ vigorous physical activity (VPA) levels, sleep latency and efficiency, and screen time compared to the control group within the Chinese cultural context.

Since parents are the primary medium through which the intervention contents were delivered in the study, the interference of parental engagement during the intervention process on preschoolers’ outcomes cannot be ruled out. Extensive studies have indicated that parents who actively interact and engage in health discussion groups—writing posts, leaving comments, asking questions, sharing personal experiences, and providing support to others (all of which are considered essential components in eHealth interventions)—gain greater benefits in terms of child behavior than passive parents [31,32,33,34].

### 1.1. Missing Link

Far too little attention has been paid to the influence of parental engagement in parent-based eHealth interventions. The characterization of varying levels of parental engagement is important to increase our understanding of tailoring further parent-based eHealth interventions and maximizing their effectiveness.

### 1.2. Objective of This Study

In the current study, we aim to determine if parental engagement in the WeChat 8.0.51 group of a parent-based eHealth intervention affected preschoolers’ PA, DB, or sleep.

### 1.3. Hypotheses

It was hypothesized that parents who engaged more actively with the social media elements of a parent-based eHealth intervention would achieve superior outcomes in their preschoolers’ PA, DB, and sleep in comparison to those who were less engaged.

## 2. Method

### 2.1. Overview

The current study utilized baseline, post-test (12 weeks after baseline), and follow-up (12 weeks after post-test measurement) data from a parent-based eHealth intervention for children aged three to six years, designed as a single-blinded RCT with two parallel arms to explore the intervention’s influence on preschoolers’ PA, DB, and sleep. Detailed information from this intervention has been previously published [29], with the intervention comprising a 12-week intervention and a 12-week follow-up. Data collection at baseline, post-test, and follow-up was conducted in four kindergartens in Guiyang, Guizhou Province, mainland China, assisted by Sun Yat-Sen University and Guiyang Preschooler Education College, between October 2023 and July 2024, with a 12-week interval between each assessment. The study was approved by the research ethics committee of Hong Kong Baptist University (SOSC-SPEH-2022-23_115) and was registered with the ClinicalTrial.gov Protocol Registration and Results System (PRS)—NCT06025019 (registered in May 2023).

### 2.2. Participants Eligibility

**Figure 1:**
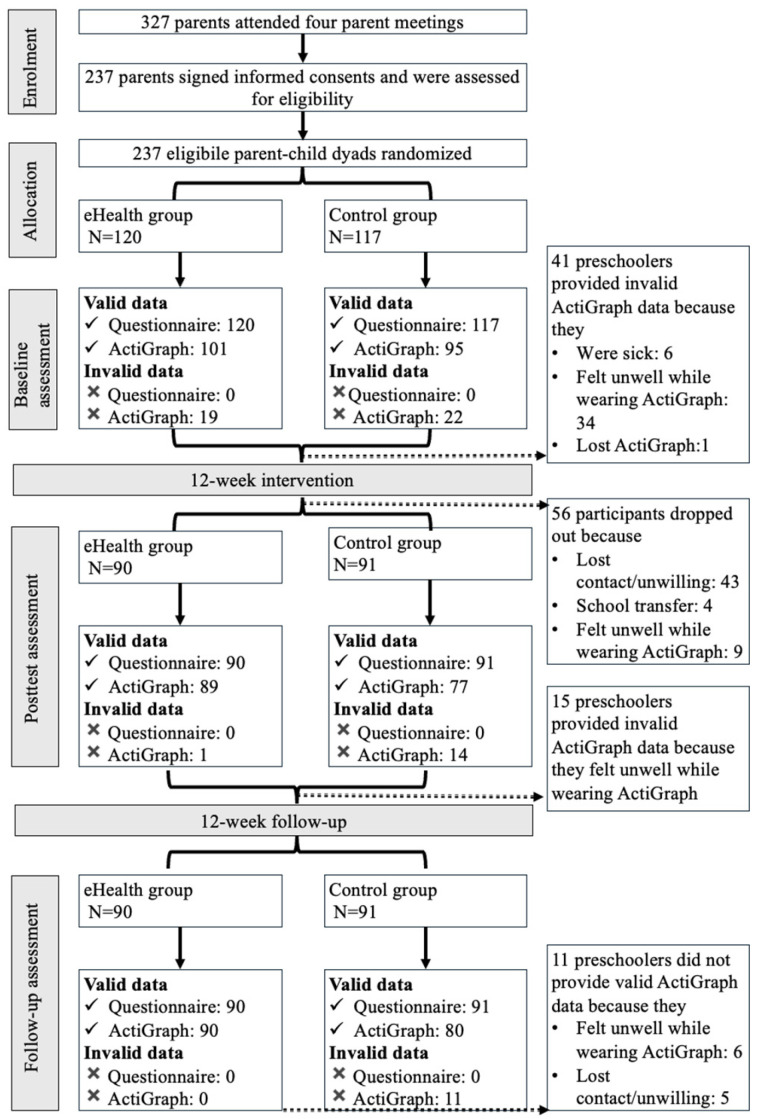
Flow chart of data collection with reason of attrition and invalid data.

The inclusion criteria were as follows: Parents needed to (1) be over 21 years old with 3–6-year-old children; (2) promise to participate in the entire 24-week intervention; (3) have access to mobile technologies; (4) be healthy and have healthy children (referring to a state of physical, mental, social, intellectual, and emotional well-being and the absence of disease) able to participate in normal PA; and (5) reside with the participating child for at least four days a week (see Figure 1).

### 2.3. Intervention Group

The parent-based eHealth intervention was designed to promote preschoolers’ PA, DB, and sleep by providing parents with relevant health information and recommendations through WeChat and motivating them to create a family environment with healthy regulations in which their children live. The relevant health information provided to parents throughout the intervention modules focused on reducing sedentary behavior and promoting physical activity, healthy eating, and sufficient sleep by explaining their developmental importance, established guidelines, and practical integration strategies—such as goal setting, replacing screen time with active play, and teaching children to choose core foods and interpret nutrition labels.

Eligible parent–child dyads allocated to the eHealth intervention group were organized into WeChat groups and provided with intervention content. Kindergarten teachers acted as facilitators in each WeChat group and were blinded to the research objectives.

The eHealth intervention comprised twelve interactive modules focused on PA, diet, and sleep, with each category containing four modules, with modules rotated weekly over the 12-week intervention period (i.e., PA, diet, sleep, PA, diet…) to mitigate the effect of order on the outcome variables. Each module consisted of following components: (1) A weekly theme-related video recorded by the researchers that lasted for 3 min and contained age-specific evidence-based recommendations, benefits of addressing the target behavior in children, common barriers, goal setting and an action plan, and feedback and monitoring protocols [35,36]. (2) Group interactions, with the facilitator of each group encouraging parents to communicate with other WeChat group members by sharing suitable PA, photos of meals or healthy snacks, and personal ideas and experiences regarding reducing sedentary behaviors and improving sleep. (3) Goal setting, with researchers informing the parents via private text message of how to help their children set PA, DB, and sleep goals. (4) A first assessment message sent by facilitators mid-week for feedback on goal progress. (5) Individualized feedback provided by researchers based on parents’ interaction in the WeChat group and goal achievement. (6) A second assessment message sent by facilitators on weekends to evaluate goal attainment before introducing a new weekly theme.

### 2.4. Control Group

Parent–child dyads assigned to the control group were invited to WeChat groups where they received electronic material related to a theme at the beginning of each week but did not receive any interactive components.

## 3. Measurements

### 3.1. Demographic Information

Parents’ information included socioeconomic status (range of income, educational level, and occupational status), age, gender, marital status, number of children, and BMI. Children’s information included age, gender, height, weight, and Body Mass Index (BMI). The detailed statistics of these measurements have been previously described [37].

#### 3.1.1. Preschoolers’ Physical Activity

PA level and sedentary time were objectively monitored using a tri-axial accelerometer ActiGraph GT3X-BT (ActiGraph, Pensacola, FL, USA). Parents were asked to record an activity diary for both wear and non-wear time, with accelerometer wear checked by teachers on each school day. The accelerometers were affixed to the children’s right wrist to monitor all activities for a period of seven continuous days, except during periods of water-related activities or situations involving a risk of damage to the device, such as swimming and bathing, with valid wear time considered to be at least 16 h over at least three days (two weekdays and one weekend day) [38]. Non-wear time was defined by 20 consecutive minutes of zero counts/minute, the standard ActiLife definition. ActiLife software (version 6.13) was used to initialize the devices and analyze the data. A recording epoch of 1 s was set for PA measurement, with accelerometers initialized at a sampling rate of 30 Hz and reintegrated into 60-s epochs for analysis. The accelerometers’ activity counts were categorized into different intensities (i.e., sedentary behavior, LPA, MPA, and VPA) using the following cut-off points: sedentary: <819 counts per minute (CPM); light: 820–3907 CPM; moderate: 3908–6111 CPM; and vigorous: ≥6112 CPM. Additionally, a log sheet was provided for parents to record when the device was removed and the reasons for removal [38]. ActiGraph accelerometers have been found to be valid and reliable in objectively measuring PA levels in preschoolers [39].

#### 3.1.2. Preschoolers’ Dietary Behaviors

Children’s dietary behaviors were assessed using the Children’s Eating Behavior Questionnaire (CEBQ), which has been examined for validity in Chinese preschool-age children [40]. Parents were asked to rate the frequency of their child’s eating behaviors (5-point scale: 1 = never, 2 = rarely, 3 = sometimes, 4 = often, 5 = always) in terms of eight domains (35 items): satiety responsiveness (SR, e.g., My child has a big appetite), slowness in eating (SE, e.g., My child finishes meal quickly), food fussiness (FF, e.g., My child refuses new food at first), food responsiveness (FR, e.g., My child is always asking for food), enjoyment of food (EF, e.g., My child looks forward to mealtimes), desire to drink (DD, e.g., My child is always asking for a drink), emotional undereating (EUE, e.g., My child eats less when angry), and emotional overeating (EOE, e.g., My child eats more when annoyed). The higher the mean score for each subscale, the more the child displays behavior tracked by that subscale. The scales have high internal consistency reliability (overall Cronbach’s Alpha above 0.7) [40].

#### 3.1.3. Preschoolers’ Sleep Quality

ActiGraph accelerometers were also used to examine children’s nighttime sleep duration, sleep latency, and sleep efficiency, in conjunction with a parent-reported log sheet. Bedtime and wakeup time were identified based on an algorithm by Sadeh, Sharkey [41], and periods of sleep were estimated using an algorithm by Tudor-Locke, Barreira [42]. Nighttime sleep duration, bedtime, and wakeup time were assessed using ActiLife software (version 6.13) in 60 s epochs and matched with the log sheets completed by parents. Sleep data was considered valid based on a wear time of at least 3 nights [43].

#### 3.1.4. Preschoolers’ Sleep Problems

Children’s sleep problems were examined by the Chinese version of the Children’s Sleep Habits Questionnaire (CSHQ) [44], a parent survey frequently used to screen children aged from 3 to 10. It contains 33 items, forming a set of eight domains: bedtime resistance, sleep onset delay, sleep duration, sleep anxiety, night waking, parasomnia, sleep-disordered breathing, and daytime sleepiness, with each question rated on a 3-point scale as “usually if something occurs 5 or more times in a week” (scored 3), “sometimes if something occurs 2–4 times in a week” (scored 2), or “rarely if something occurs never or 1 time during a week” (scored 1); a total score of over 41 demonstrates a sleep disorder. The CSHQ has shown good reliability and validity (Cronbach’s Alpha of 0.73) [44].

#### 3.1.5. Preschoolers’ Screen Time

Parents were asked to answer questions which estimate the usual amount of screen time for their children on a typical weekday and weekend to determine the average screen time per week, with the questions also considering the availability of screens and rules about screen entertainment. This questionnaire was used in a previous study and back-to-back translated into Chinese [43]. The questionnaire includes the following four questions: “Do you have rules regarding screen time at home?” (A) Yes, (B) No. “Does your child’s bedroom have any screen devices?” (A) No, (B) Yes. In these two questions, A earns one point and B earns two. “How often does your child watch TV while eating?” (A) Never, (B) 1–3 times a week, (C) 4–6 times a week, (D) 7 times a week or more. Here, A earns one point; B, two; C, three; and D, four. “On average, how much time does your child spend in front of a screen on weekdays (Monday to Friday) and on weekends each day?”

### 3.2. Statistical Analysis

Because we aimed to investigate the impact of parental engagement in an eHealth intervention on preschoolers’ PA, DB, and sleep, only the data collected from the intervention group at the three assessment points was used.

All statistical analyses were conducted using IBM SPSS 29, and a two-sided *p* value < 0.05 was considered statistically significant. Descriptive statistics (mean ± SD for continuous variables, number and percentage for categorical variables) are presented for demographic information and outcome variables. Parents were categorized in two groups based on how they interacted with the weekly theme through the WeChat groups or private messages with the researchers. (1) The lurker group refers to participants who only responded to the weekly self-assessment messages, but aside from this did not interact within WeChat groups or message the researchers privately. (2) The actively engaged group referred to participants who, in addition to responding to the weekly self-assessment messages, not only actively posted photos of their children participating in PA and healthy eating, but also shared strategies for improving sleep routines and reducing sedentary behaviors at least once a week, either in the WeChat group or through private messages with the researchers. This category aligns with the definition of ‘active’ engagement used in a similar study [45]. The entire parental engagement coding process was conducted by two researchers who were not involved in this study and not aware of the research objectives. The first researcher grouped parents based on the frequency of their messages in the WeChat group and private messages with the research staff throughout the 12-week period; the second researcher verified the grouping results to ensure the accuracy of classification into the lurker and actively engaged groups.

A power analysis using G*Power 3.1.9.6 [46] indicated that for a two-group repeated measures ANOVA with three measurements and an expected effect size of 0.20, significance level of 0.05, desired power of 0.80, correlation between repeated measures of 0.5, and nonsphericity correction of 1 applied, the total sample size should be 42, with 21 participants allocated to each group [31,47].

Data collected from wGT3X-BT ActiGraph and parent-reported questionnaires were analyzed by Generalized Estimating Equations (GEEs), using group and time as the main effects and by adjusting demographic information for covariates (i.e., preschoolers’ gender, age, height, weight, and BMI; parents’ age, height, weight, gender, educational level, income range, marital status, and BMI; and number of children in the household) to examine the effects of parental engagement in a parent-based eHealth intervention on preschoolers’ PA, DB, and sleep. A pairwise comparison was conducted using Bonferroni adjustment for multiple comparisons to compare the differences among four groups whenever a group*time interaction effect was observed. Missing values were not imputed as the GEEs provide a regression framework for handling missing data in an appropriate way [48].

## 4. Results

### 4.1. Baseline Characteristics of Participants Allocated to the eHealth Intervention Group

A total of 327 parent–child dyads were initially interested in the eHealth intervention study, with 237 parent–child dyads deemed eligible after screening, of which 120 were randomly assigned to the intervention group.

At the baseline assessment, 120 parents completed the questionnaires and 101 preschoolers provided valid ActiGraph data. Post-test, 90 parents completed the questionnaires and 89 preschoolers provided valid ActiGraph data. At the follow-up assessment, 90 parents completed the questionnaires and 90 preschoolers provided valid ActiGraph data.

For the preschoolers, the mean age (±SD) was 4.51 (±0.72) years, and 55.8% were boys. The mean BMI (±SD) was 16.50 ± 3.74.

The mean age (±SD) of the parents was 34.28 (±4.74) years, and 77.5% of the involved parents were mothers. The parents’ mean BMI (±SD) was 22.59 ± 5.29, and 25% had only one child, 56.7% had a bachelor’s degree, and 4.2% were divorced/separated, while 60% of families made 7346–20,000 CNY/month. The baseline characteristics of those assigned to the actively engaged group and lurker groups are displayed in Table 1; detailed demographic information can be found elsewhere [37].

### 4.2. Parental Engagement in WeChat Groups

Parents assigned to the intervention condition were organized into fourteen nine-member WeChat groups, with group size based on the optimal group interactive effect [49]. Sixty-seven parents (56%) did not interact at all throughout the intervention period and were thus allocated to the Lurker group, while fifty-three parents (44%) shared photos or posted strategies to improve PA, diet, sleep, and screen time once a week, and were thus classified into the actively engaged group (Table 1). Only a small portion of parents interacted more than once a week, approximately fifteen parents, accounting for 12.5% of the total.

### 4.3. The Influence of Parental Engagement on Preschoolers’ Behavioral Change

Table 2 presents the GEE results assessing between-group differences over time by the level of parental engagement on WeChat.

Compared to preschoolers of parents who did interact in WeChat throughout the 12-week intervention, those with actively engaged parents showed dramatically increased MVPA (adjusted mean difference: 581.73 min/7 days, *p* = 0.000) and VPA (adjusted mean difference: 596.27 min/7 days, *p* = 0.000) and significantly decreased weekend screen time (adjusted mean difference: −26.14 min/day, *p* = 0.000) at the post-test.

In addition, at the post-test, children with parents in the actively engaged group showed decreases of 0.37 in satiety responsiveness (*p* = 0.007), 0.30 in desire to drink (*p* = 0.003), and 0.28 in food fussiness (*p* = 0.011) compared to those in the lurker group.

The different levels of parental engagement showed no significant effect on sleep-related outcomes in preschoolers.

## 5. Discussion

Social-media-integrated interventions aimed at modifying health behaviors in preschoolers through parental involvement hold significant promise, as social media fosters supportive communities where parents can share experiences and receive immediate feedback, enhancing motivation and commitment to health behavior changes in a cost-effective manner [50]. Although several attempts have been made to use social media platforms—such as Facebook and WhatsApp—as intervention delivery methods to enhance healthy lifestyle behaviors [25,49,51], these studies measured parental engagement using retention, attendance, and percentage of intervention modules and goals completed [52,53,54], meaning the literature addressing the impact of different parental engagement levels on PA, diet, and sleep in early childhood is limited. This study aimed to determine whether parental engagement via WeChat, as an integral part of a parent-based eHealth intervention, affected preschoolers’ health outcomes. Overall, the results indicated that, compared to the preschoolers with less-engaged parents, those whose parents posted photos and comments related to interactive themes or shared strategies to improve children’s healthy lifestyle behaviors through WeChat significantly improved in terms of PA, diet, and screen time after a 12-week intervention. However, these changes were not sustained at follow-up.

### 5.1. The Impact of Parental Engagement on Preschoolers’ PA

Active parental participation in the WeChat component of the intervention could be a factor contributing to preschoolers’ improved MVPA and decreased sedentary behavior at post-test in comparison to those with lurker parents. The observed increased MVPA and decreased sedentary behavior may be because of the 24 h activity cycle, with the distribution of a given 24 h period among the interdependent and inseparable states of sleep, sedentary behavior, and PA—on a continuum from no movement to high movement—meaning that when the rate of one behavior increases, the others decrease [55]. The possible impacts of social interaction and encouragement from other parents in WeChat groups cannot be ruled out, which may enhance intrinsic motivation and make parents more likely to implement PA-promoting strategies for their children [56]. Engaged parents feel a stronger sense of accountability due to their involvement in discussions and activities, and by participating actively, they serve as role models, demonstrating the value of PA and motivating their kids to be more active. Additionally, this active involvement fosters a sense of community among parents, providing social support that helps them sustain and promote healthy behaviors at home [57].

The finding is contrary to that of Hammersley, Okely [31], who reported an inverse relationship between active Facebook engagement and preschoolers’ MVPA (estimate, −0.14; 95% CI, −0.26 to −0.01; *p* = 0.03) at post-test. Monitoring parental engagement via the frequency of parents “liking”, “posting”, and “commenting” within a Facebook group, the study [31] compared the effects of actively engaged parents (those who made both comments and posts on the respective module) with passively engaged parents (those who either did not post or comment, or only liked the posts) on preschoolers’ lifestyle behaviors [31]. The inconsistent finding could be attributed to the study’s sample size, as throughout the 11-week intervention, there was only one module focused on PA, with only 4 of the 42 intervention-group parents posting about the module and 9 parents commenting. These notably low levels of active engagement observed could contribute to a Type II error. Very small samples undermine both the internal and external validity of a study, limiting the study’s ability to detect significant effects and failing to identify the true impact of parental engagement on preschoolers’ PA [58]. A possible explanation for our findings’ misalignment with those of Hammersley, Jones [43] could be module ordering, with their study only implementing six models—one delivered every two weeks—meaning the PA module was conducted over a month before the post-test. Parents may have been highly engaged during the PA module, but the time elapsed between the module and the post-test could have led to a decrease in their influence on preschoolers’ MVPA as they potentially lose motivation or forget strategies discussed during the PA module, leading to reduced reinforcement of active behaviors in their children.

### 5.2. The Impact of Parental Engagement on Preschoolers’ Diet

At post-test, significant changes were observed in satiety responsiveness, desire for sweet beverages, and food fussiness in preschoolers with actively engaged parents in comparison to those with lurker parents, aligning with the hypothesized direction based on engagement frequency. During the intervention, parents learned four key knowledge points in the diet modules: (1) what should be included in a preschooler’s daily diet, emphasizing the importance of vegetables, fruits, grains, and proteins; (2) how to address picky eating and ensure a balanced nutrient intake; (3) the recommended standards for salt and sugar consumption in daily life and the risks of exceeding these limits; and (4) healthy feeding practices for parents. Through interactions with other parents, actively engaged parents enhanced their knowledge about nutrition and healthy eating practices, increasing awareness and potentially leading parents to expose their children to a wider variety of foods, thereby fostering positive food behaviors, including a decreased desire to drink and reduced food fussiness.

Additionally, many Chinese parents hold a concept that larger body weight and size are indicators of healthy growth, associating being overweight with affluence [59]. As a result, they may prefer their children to appear chubbier and underestimate the implications of overweight or obesity [60]. This perspective could lead actively engaged parents to pressure their children to eat more, which has been shown to correlate with heightened enjoyment of food, decreased satiety responsiveness, and increased food fussiness [60].

Furthermore, entrenched food-related cultural norms in Chinese parenting could further diminish preschoolers’ satiety responsiveness and fussiness. These practices are often rooted in values that emphasize not wasting food and ensuring children’s nutritional needs are met [61]. Parents frequently serve large portions as a way to express love and care, encouraging their children to finish everything on their plates, regardless of hunger cues [62]. Over time, such practices can reinforce behaviors that lead children to eat beyond their physiological needs, resulting in lower satiety responsiveness and increased food fussiness [62].

### 5.3. The Impact of Parental Engagement on Preschoolers’ Sleep

The lack of significant effects of varying parental engagement levels on preschoolers’ sleep-related outcomes can be attributed to several factors. Firstly, sleep is influenced by a range of biological, environmental, and psychological factors beyond parental involvement, including genetics, health status, and the sleep environment [63], and the active engagement of parents during the 12-week intervention may not have been enough to lead to changes in their children’s sleep outcomes. Additionally, preschoolers may have already established sleep patterns that are resistant to change, particularly if these habits have been ingrained over time [64]. Lastly, in the context of the Chinese labor market, many parents experience long working hours and unstable, unpredictable schedules (such as rotating shifts), which can limit their presence at home [65]. This situation can disrupt regular family routines and make it challenging to establish consistent household regulations, including sleep routines. Consequently, even if parents actively participated in the intervention, their absence might hinder their ability to effectively influence their children’s sleep behaviors.

### 5.4. Practical Implications

Our findings offer several evidence-based insights for the design and implementation of digital health interventions targeting early-childhood behaviors. Primarily, the dramatic increases in children’s MVPA and VPA among the actively engaged group suggest that the depth of parental interaction is more critical than mere enrollment; therefore, future interventions should prioritize strategies—such as interactive prompts or gamified rewards—that transition parents from passive “lurking” to active participation. Furthermore, the significant improvements in dietary behaviors (e.g., reduced food fussiness and satiety responsiveness) and weekend screen time indicate that active digital engagement effectively translates into home-based behavioral changes, particularly when targeting vulnerable periods like weekends. However, the disparity in health outcomes between “active” and “lurker” groups highlights a need for early detection systems to provide personalized outreach or “booster” interventions for less-engaged participants. Finally, the lack of significant impact on sleep-related outcomes suggests that general digital social interactions may be insufficient for sleep modifications, requiring practitioners to consider more specialized or intensive support models to address the complexities of preschoolers’ sleep.

## 6. Limitations

The preschoolers in the present study were from an economically undeveloped region in China; thus, our findings are limited to a specific population. Additionally, our sample size was relatively small and we only analyzed the frequency of interactions related to each weekly theme, hampering our ability to determine which specific interaction methods contributed to behavioral changes in preschoolers. This study is limited by parental self-selection and the resulting group imbalance. As participants were not randomized into ‘active’ or ‘lurker’ groups—rather, these categories emerged based on natural usage patterns—self-selection bias cannot be ruled out, as parents who engaged more actively may have been more motivated or had more resources to implement behavioral changes in their children. This imbalance in group size and characteristics may limit the reach of the findings and suggest that the observed benefits in the engaged group could be partially attributed to pre-existing parental factors. Future studies should consider strategies to encourage uniform engagement or use randomized designs to better isolate the effects of digital interaction.

## 7. Conclusions

This study investigated the impact of varying levels of parental engagement in the WeChat component of a parent-based eHealth intervention on preschoolers’ PA, DB, and sleep. The findings revealed that actively engaged parents within WeChat (i.e., posting and commenting at least once a week about the corresponding modules) significantly increased preschoolers’ MVPA and reduced sedentary behaviors, as well as decreased satiety responsiveness, desire to drink, and food fussiness compared to children whose parents were passively engaged. Further research is needed with larger sample sizes and longer duration to better investigate the potential of social media in parent-based interventions for promoting healthy lifestyles in children.

## Figures and Tables

**Table 1:** Baseline demographic information of participants in the two groups.

Demographic Characteristics		Actively Engaged Group (n = 53)	Lurker Group (n = 67)	*p* Value
Preschoolers				
Age (years) (mean ± SD)		4.36 ± 0.27	4.12 ± 0.51	0.555
Gender (number)	Boy	30	24	0.070
	Girl	23	43	
Height (meters) (mean ± SD)		1.07 ± 0.02	1.10 ± 0.02	0.723
Weight (kg) (mean ± SD)		19.38 ± 4.85	19.74 ± 4.95	0.552
BMI (kg/m^2^) (mean ± SD)		16.42 ± 3.68	16.58 ± 3.80	0.732
Overweight/obese (number)		10	16	0.110
Parents				
Age (years) (mean ± SD)		34.12 ± 4.68	34.44 ± 4.81	0.672
Gender (number)	Mother	43	50	0.053
	Father	10	17	
Height (meters) (mean ± SD)		1.60 ± 0.06	1.62 ± 0.06	0.076
Weight (kg) (mean ± SD)		58.12 ± 14.25	59.86 ± 14.52	0.513
BMI (kg/m^2^) (mean ± SD)		22.42 ± 5.15	22.72 ± 5.40	0.755
Overweight/obese (number)		24	20	0.450
Only one child (number)		12	18	0.051
Marital status	Married	50	65	0.111
	Divorced	3	2	
Education	College and below	32	20	0.330
	Bachelor’s	20	26	
	Master’s and above	6	6	
Income (RMB per month) (number)	RMB ≤ 3000	2	2	0.060
	3001 < RMB ≤ 7345	17	13	
	7346 < RMB ≤ 20,000	42	30	
	RMB > 20,000	9	5	

**Table 2:** Summary of post hoc analysis of Generalized Estimating Equations (GEEs) whenever a group × time effect was detected (n = 120).

Actively Engaged Group vs. Lurker Group				
Variables	Post-Test		Follow-Up Test	
	Adjusted Mean Difference (95% CI)	*p* Value	Adjusted Mean Difference (95% CI)	*p* Value
LPA (min/7 days)	−134.54 (−364.19, 95.10)	0.442	46.16 (−197.77, 290.10)	1.000
MPA (min/7 days)	−14.54 (−119.74, 90.67)	0.130	61.79 (−75.11, 198.69)	0.778
Toal MVPA (min/7 days)	581.73 (264.82, 898.63)	0.000	78.20 (−54.07, 210.47)	1.000
VPA (min/7 days)	596.27 (274.61, 917.92)	0.000	17.57 (−33.51, 68.65)	1.000
Enjoyment of food (questionnaire score)	0.04 (−0.46, 0.53)	1.000	0.07 (−0.43, 0.57)	1.000
Emotional overeating (questionnaire score)	0.05 (−0.33, 0.43)	1.000	−0.09 (−0.32, 0.13)	1.000
Satiety responsiveness (questionnaire score)	−0.37 (−0.79, 0.01)	0.07	0.09 (−0.26, 0.45)	1.000
Slow eating (questionnaire score)	−0.18 (−0.68, 0.33)	1.000	−0.15 (−0.63, 0.33)	0.592
Desire to drink (questionnaire score)	−0.30 (−0.53, −0.06)	0.003	0.02 (−0.59, 0.62)	1.000
Food fussiness (questionnaire score)	−0.28 (−0.53, −0.04)	0.011	−0.08 (−0.39, 0.22)	1.000
Emotional undereating (questionnaire score)	0.03 (−0.39, 0.46)	1.000	0.08 (−0.27, 0.42)	1.000
Food responsiveness (questionnaire score)	−0.14 (−0.59, 0.30)	1.000	−0.02 (−0.46, 0.39)	1.000
Sleep problems (questionnaire score)	−1.61 (−6.02, 2.81)	0.578	−2.89 (−6.79, 1.00)	0.441
Sleep latency (min/day)	1.04 (−0.87, 2.95)	0.134	0.52 (−2.36, 3.39)	1.000
Sleep efficiency (%)	4.00 (−11.26, 19.27)	1.000	−3.22 (−17.88, 11.43)	1.000
Sleep duration (min/day)	−5.19 (−45.98, 35.60)	1.000	2.02 (−20.41, 24.45)	1.000
Screen time in weekdays (min/day)	−1.53 (−13.14, 16.21)	1.000	−4.95 (−13.20, 23.09)	0.41
Screen time in weekend days (min/day)	−26.14 (−32.71, −19.56)	0.000	4.49 (−6.26, 15.24)	0.335

Abbreviations: LPA: light physical activity; MPA: moderate physical activity; MVPA: moderate-to-vigorous physical activity; VPA: vigorous physical activity; CI: confidence interval. Bonferroni correction was used to adjust for multiple pairwise comparisons.

## Data Availability

The datasets generated and/or analyzed during the current study are not publicly available due to participant privacy and ethical restrictions, but are available from the corresponding author on reasonable request.
